# His Bundle Activates Faster than Ventricular Myocardium during Prolonged Ventricular Fibrillation

**DOI:** 10.1371/journal.pone.0101666

**Published:** 2014-07-18

**Authors:** Nathan Angel, Li Li, Derek J. Dosdall

**Affiliations:** 1 Comprehensive Arrhythmia Research & Management Center, Division of Cardiovascular Medicine, University of Utah, Salt Lake City, UT, United States of America; 2 Department of Bioengineering, University of Utah, Salt Lake City, UT, United States of America; 3 Center for Engineering Innovation, University of Utah, Salt Lake City, UT, United States of America; University of Minnesota, United States of America

## Abstract

**Background:**

The Purkinje fiber system has recently been implicated as an important driver of the rapid activation rate during long duration ventricular fibrillation (VF>2 minutes). The goal of this study is to determine whether this activity propagates to or occurs in the proximal specialized conduction system during VF as well.

**Methods and Results:**

An 8×8 array with 300 µm spaced electrodes was placed over the His bundles of isolated, perfused rabbit hearts (n = 12). Ventricular myocardial (VM) and His activations were differentiated by calculating Laplacian recordings from unipolar signals. Activation rates of the VM and His bundle were compared and the His bundle conduction velocity was measured during perfused VF followed by 8 minutes of unperfused VF. During perfused VF the average VM activation rate of 11.04 activations/sec was significantly higher than the His bundle activation rate of 6.88 activations/sec (p<0.05). However from 3–8 minutes of unperfused VF the His system activation rate (6.16, 5.53, 5.14, 5.22, 6.00, and 4.62 activations/sec significantly faster than the rate of the VM (4.67, 3.63, 2.94, 2.24, 3.45, and 2.31 activations/sec) (p<0.05). The conduction velocity of the His system immediately decreased to 94% of the sinus rate during perfused VF then gradually decreased to 67% of sinus rhythm conduction at 8 minutes of unperfused VF.

**Conclusion:**

During prolonged VF the activation rate of the His bundle is faster than that of the VM. This suggests that the proximal conduction system, like the distal Purkinje system, may be an important driver during long duration VF and may be a target for interventional therapy.

## Introduction

Sudden cardiac death due to ventricular fibrillation (VF) is one of the leading causes of death in the developed world[1]. Furthermore, sudden cardiac death is often unexpected, occurring without any predetermined risk[2]. Implantable cardiac defibrillators (ICD’s) have greatly reduced mortality due to VF, likely due to defibrillation shocks being applied to the myocardium soon after the onset of VF[3]. However this treatment is only available for those that have been clinically diagnosed as high risk for VF. For those without ICD’s, VF often persists for 5–10 minutes before defibrillation shocks can be applied[4]. Since the probability of successful resuscitation from VF decreases 10% for every minute of VF, the survival rate for out of hospital sudden cardiac arrest is low[5]. Successful resuscitation rate is thought to decrease during prolonged VF because of electrophysiological changes to cardiac tissue under ischemic conditions[6]. These electrophysiological changes may cause permanent cardiac damage[7] and can result in failure to defibrillate successfully[8].

The Purkinje fiber system has recently been implicated as an important driver of VF activation in the maintenance of long duration VF (VF>2 min) [9]–[11]. Distal Purkinje fibers have been shown to develop triggered activity during VF due to the ischemic conditions which is thought to drive this rapid activation[6], [12]. However it is unclear whether this rapid activation rate is limited only to the distal regions of the conduction system, or whether it is observed or conducts to the proximal regions of the conduction systems as well.

Due to the several minute delay of therapy for patients after the onset of VF, it is necessary to study the electrophysiological properties of the ventricular conduction system during prolonged VF to aid in the development of therapies to improve patient outcome. In this study, electrophysiological properties of the conduction system of interest are the activation rate of the His bundle compared to the VM and the His bundle conduction velocity during the time course of VF. The ventricular conduction system has a much higher conduction velocity than the VM and only interacts with the VM at specialized junctions known as the Purkinje-myocardial junctions (PMJs) [13]. Due to the faster conduction velocity and limited access sites into the ventricular conduction system from the myocardium, activation in the distal Purkinje system may travel quickly throughout the conduction system. We tested the hypothesis that the proximal regions of the ventricular conduction system, like the distal conduction system, have a faster activation rate than the underlying ventricular myocardium (VM) during prolonged VF.

## Methods

All animals were managed in accordance with the *Guide for the Care and Use of Laboratory Animals* [14], and the protocol was approved by the Institutional Animal Care and Use Committee of the University of Utah. All efforts were made to minimize suffering.

### Animal Preparation

Twelve male New Zealand white rabbits (3.9±0.2 kg) were anesthetized with an intramuscular injection of 15 mg/kg ketamine, and 7.5 mg/kg xylazine followed by an intravenous injection of 5 mg/kg ketamine, 2.5 mg/kg xylazine, and 500 IU of heparin. The hearts were excised rapidly and Langendorff-perfused with Tyrode solution (in mmol/L 130 NaCL, 1.2 NaH_2_PO_4_, 1 MgCl_2_, 4 KCl, 1.8 CaCl_2_, NaHCO_3_ 20.8, dextrose 11, and 0.04 g/l bovine albumin). Flow rate was adjusted to maintain a pressure at 25±3 mmHg. The hearts were also superfused with warm Tyrode solution, with temperature maintained at 37±0.5 °C. The Tyrode solution was oxygenated with O_2_ and CO_2_ to maintain a pH of 7.4±0.1.

### Instrumentation and Experimental Protocol

An incision in the right atrium was made to expose the His bundle, which is found along the atrial-ventricular boundary high on the RV septum. Unipolar VM and His bundle activity were recorded using an 8×8-electrode plaque with electrodes spaced 0.3 mm apart with a reference electrode in the solution bath. Electrograms were low pass filtered at 1640 Hz, sampled at 8 KHz, and recorded at 24 bit resolution (Active Two System, Biosemi, Inc, Amsterdam, Netherlands). Electrodes were placed in the perfusate on either side of the heart (one near the atrium and the other near the ventricle) to calculate a bipolar pseudo-ECG. VF was induced with 15–30 seconds of 50 Hz burst pacing, with amplitude of 5 mA through bipolar leads placed in the right ventricular apex. After 30 seconds of sustained VF, perfusion was stopped and VF persisted until it spontaneously terminated. The electrode plaque was lifted off of the myocardium and His bundle using a micromanipulator every 30 seconds to avoid ischemia of the His bundle.

### Measurements and Data Analysis

His, VM, and atrial myocardium (AM) activations were differentiated by calculating a 5-point Laplacian from the unipolar electrograms. The Laplacian signals were calculated according to Punske et al. [15]. In brief, a standard central difference approach was used as shown in the formula,
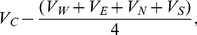
(1)where *V* is the voltage, subscript C refers to the central electrode, and the other subscripts, W, E, N, and S, refer to the relative positions of the other electrodes surrounding the central electrode according to the compass regions. If the electrode site was on the corner or edge of the array, values from the two or three nearest sites were averaged and subtracted from the central electrode site.

Channels that contained strong His and VM activations were used for the analysis of activation rate and conduction velocity. An activation rate for the His and VM was determined by averaging the cycle length of the first 10 successive activations of each minute, up to 8 minutes, of VF. Activations times were determined using a thresholding approach with manual oversight via custom software developed in MATLAB (Mathworks, Inc., MA, USA). This software computed a 21-point temporal derivative of the Laplacian. During sinus rhythm, the local maximums of this filtered signal were taken as the activation times. Activation times during VF were determined by finding local maximum that had a peak that was at least 50% of the sinus rhythm local maximums. An activation map for each of the 10 His activation used to calculate the activation rate was made to determine His conduction velocity. For each of the 10 activation maps a His conduction velocity was calculated by subtracting the activation times from the most proximal and distal activation along the His bundle fiber, then dividing by the distance between those electrodes. The average of these 10 His conduction velocities were then taken as the conduction velocity for sinus, perfused, and for the 8 minutes of unperfused VF. The data are publically available at USPACE (http://content.lib.utah.edu/cdm/ref/collection/uspace/id/9710).

### Statistical Analysis

Results are expressed as mean±SD. The overall difference between VM and His bundle activation rate and the effect of time on His bundle conduction velocity was tested with repeated-measure ANOVA (XLSTAT Version 2014. 1.08). Differences between the activation rate of the VM and His bundle for perfused VF and each minute of unperfused VF were tested using a post-hoc paired *t*-test with a Holm-Bonferroni correction for multiple comparisons. His sinus conduction velocity was compared with unperfused VF and 1–8 minutes of VF with an unpaired *t*-test with a Holm-Bonferroni correction for multiple comparisons. The null hypothesis of no difference for all tests was rejected if the probability value was less than 0.05.

## Results

Hearts in which unperfused VF persisted less than 2 minutes were excluded from the analysis (n = 2). The average duration of sustained VF was 9.13 minutes±4.52 minutes (n = 10). For sinus rhythm, perfused VF, and for minutes 1–4 of VF, data from 10 hearts was analyzed. VF spontaneously terminated as time progressed so that at 5 minutes, n = 8, at 6 minutes n = 5, and at 7–8 minutes, n = 6. At 6 minutes of unperfused VF, a time point was excluded from the analysis because VF spontaneously converted to VT for 30 seconds then converted back into VF. In one heart, unperfused VF self-terminated during the 6th minute but was burst paced for 10 seconds, which reinitiated VF for another 13 minutes.

**Figure 1:**
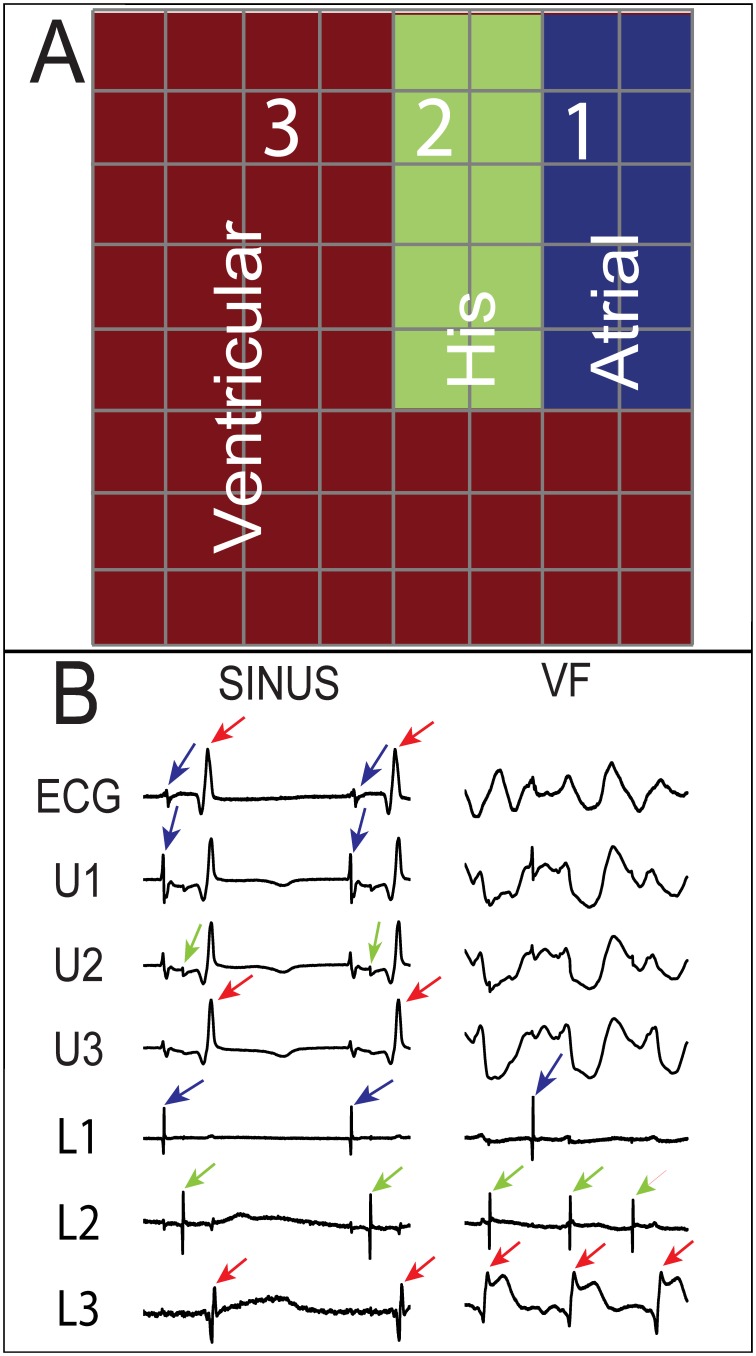
Recordings from an array placed over the His bundle of an isolated rabbit heart. **A)** Strong ventricular, His, and atrial signals were recorded during sinus rhythm from the 3 regions of the 8×8 array, 300 µm spaced electrodes, shown in red, green, and blue, respectively. Electrograms at sites 1, 2, and 3 are shown in **B)** A pseudo ECG, unipolar electrogram (from site 2 in **A**), and 3 Laplacian recordings (from sites 1, 2, and 3 in **A**) during sinus and VF. Each recording was 500 ms in duration. The unipole at site 2 shows ventricular (red arrows), His (green arrows), and atrial (blue arrows) deflection, while the Laplacians isolate the strongest local signal and eliminate far field signals. During VF, it is difficult to distinguish different activation types with the unipolar signal, but the different Laplacians facilitate waveform identification.

Figure 1A is a representative example from one experiment of the location of the three primary cardiac signals (VM, AM and His) recorded on the electrode plaque once the Laplacian had been computed. The His bundle signals were always in a thin line, (2 to 3 electrodes wide, by 3 to 5 electrodes long), in between predominate AM and VM signals. Figure 1B shows example unipolar and Laplacian recordings of the AM, VM and His signals from the array.

**Figure 2:**
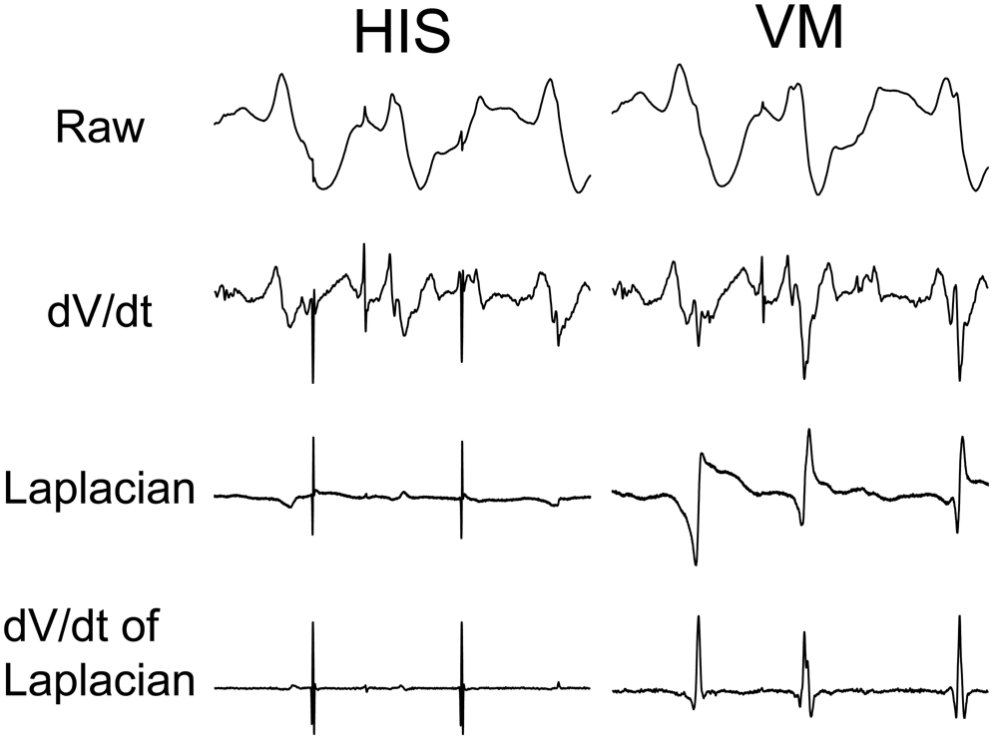
Signal processing to detect activation times for His and VM signals. His and VM signals were recorded at the location 2 and 3 from Figure 1A, respectively. The raw electrograms contain little information to detect activation times. The 21-point dV/dt of the electrograms shown above contains too much far field activation for robust local activation time detection. The Laplacian isolates the strongest local signal, attenuating far field signals. With baseline noise the zero crossing is not always clear therefore the 21-point derivative of the Laplacian was calculate which produced clear peaks, which allowed for activation detection with a thresholding approach to detect activation times. Each recording was 500 ms in duration.

Figure 2 shows the processing of electrograms to determine the activations rates for the His bundle and the VM. The raw electrograms contain little information about the activation times of the tissue. Using the minimums of the derivative is a standard method to detect activation times of cardiac tissue, however it is still not clear whether the activations are local or from far field signals. Furthermore the temporal derivative often produces multiple local minimums, making the choice of the activation time ambiguous. Due to these limitations, distinguishing His-bundle and VM activation during VF was difficult with the unipolar electrograms and derivatives. In theory, Laplacian signal shows activations at the zero crossing, however with baseline noise the zero crossing was not always clear therefore further processing was required. The 21-point temporal derivative of the Laplacian produced unambiguous peaks which were used to find activation times for the His and VM.

**Figure 3:**
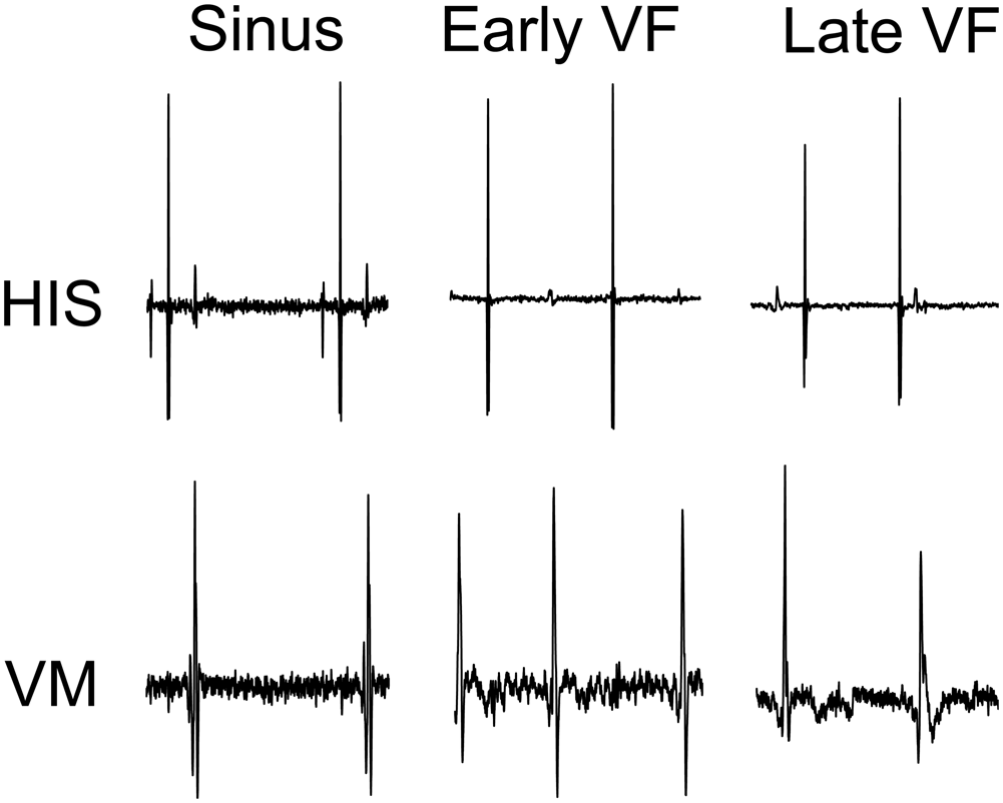
His and VM dV/dt of Laplacians during time course of VF. Early VF recording during perfused VF. Late VF is after 4

Figure 3 shows example recordings of the filtered His bundle and VM signals for sinus, early and late VF. The local maximums in the signals shown in Figure 3 were the activation times used to calculate an activation rate for the His bundle and VM and conduction velocity for the His bundle. During sinus rhythm the His bundle and VM activations were coupled because the His system activates the VM. Conversely, the His bundle and VM activations became uncoupled during VF.

**Figure 4:**
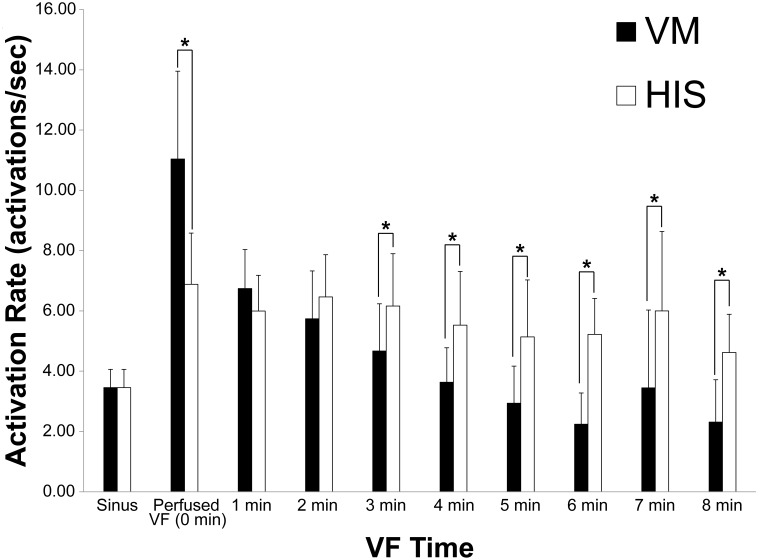
Mean and standard deviation of His and VM activation rate during eight minutes of unperfused VF. The average sinus rate for the rabbits was 3.46±0.61 activations/sec for both the His bundle and VM. *denotes p<0.05.

The repeated-measures ANOVA showed a statistically significant effect of cell type (His or VM) on activation rate and that time had a significantly effect on VM activation rate. However, time did not have a statistically significant effect on His bundle activation rate over the 8 minute time course of VF. This is shown by the similar activation rate of the His bundle during the eight minutes of unperfused VF. The activation rate for the His bundle and VM are shown as a function of time for both the His and VM with the error bars reporting the standard deviation of the measurement, representing the differences among animals (Figure 4). During perfused VF, at time 0, the average VM activation rate was significantly higher than the His rate. At 1–2 minutes there is no significant difference between VM and His activation rates, however at the 3–8 minutes the His bundle activates faster than the VM.

**Figure 5:**
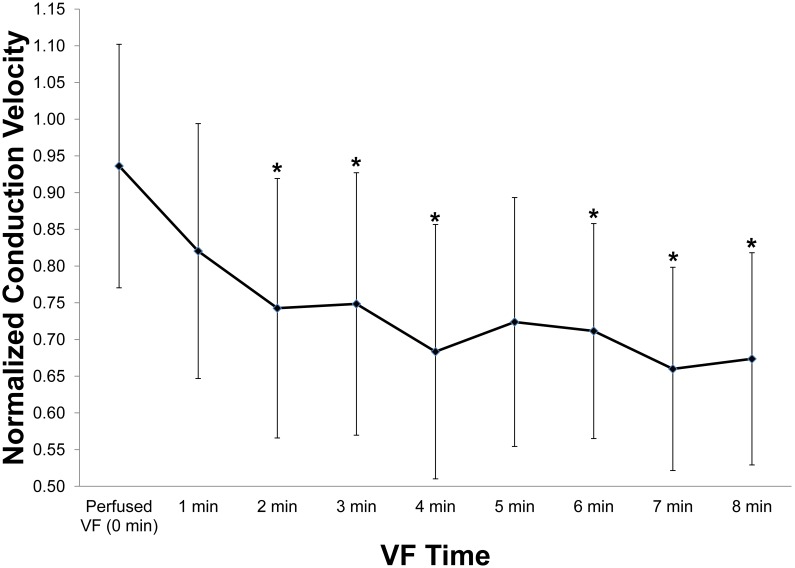
Mean and standard deviation of His conduction velocity as a function of VF duration. Values were normalized to mean His sinus conduction velocities. A repeated-measures ANOVA analysis showed that time has a significant effect on His Bundle conduction velocity. *denotes a significant difference (p<0.05) between non-normalized sinus conduction and the corresponding time point.

**Figure 6:**
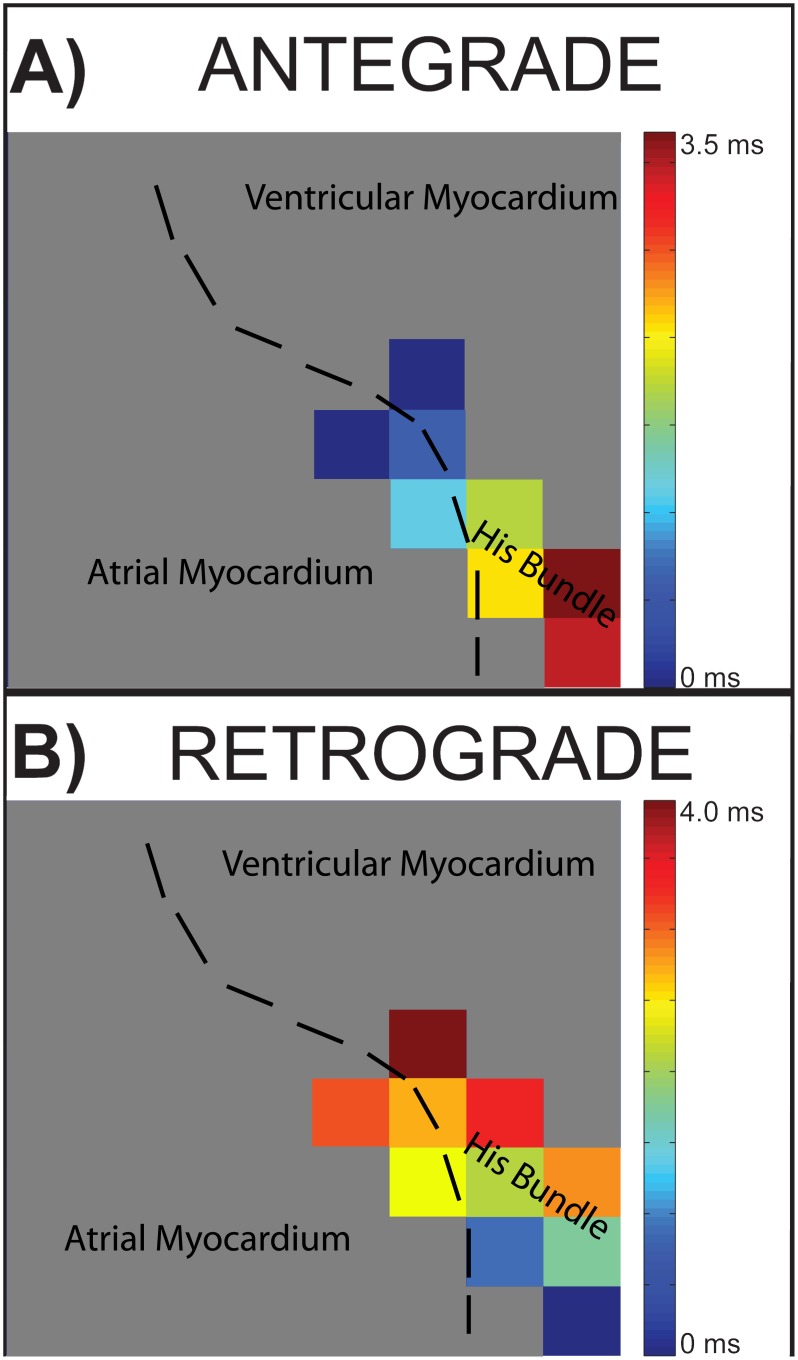
Example of His bundle antegrade and retrograde conduction. The plaque shown is a 8×8 array with 300 um electrode spacing, which covers a 2.1 mm x 2.1 mm region of the endocardium. Regions not containing strong His activity are shown in gray. **A)** Conduction during sinus rhythm was in the antegrade direction. **B)** Conduction in the His bundle during VF was almost always in the retrograde direction. The example shown is during perfused VF.

Time had a statistically significant effect on His conduction velocity. The His conduction velocity decreased from 94% at perfused VF to 67% of the sinus conduction velocity at 8 minutes of unperfused VF (Figure 5). The His conduction velocity at unperfused VF, 1 minute and 5 minutes of VF was not significant different than sinus His conduction velocity. 5 minutes of VF trended towards significance, with a corrected p-value of 0.053, and likely was not significant due to the large standard deviations. The sinus rate was 0.46±0.08 m/s with n = 10. The conduction direction in the His bundle was antegrade, His bundle to VM, during sinus rhythm and nearly entirely retrograde, VM to His bundle, during VF (Figure 6) (1 out of 650 examined activations were antegrade during VF).

## Discussion

The main findings of this study are as follows: 1) Calculating a Laplacian from unipolar electrograms aids in the identification of His-Purkinje signals. 2) The His bundle activation rate does not significantly change over the time course of VF. 3) During early VF, the VM has a higher activation rate than the His bundle, but from 3 minutes on, this relationship reverses, and the His bundle activates faster than the VM. 4) Conduction in the His bundle is nearly entirely retrograde during VF and conduction velocity gradually decreases during the time course of VF.

Laplacian signals have been used previously to accurately detect activation times during VF[15], [16]. Unique to this study is the utilization of Laplacian signals to distinguish local His bundle activations from the underlying VM activations. The traditional method to detect activation within cardiac tissue is to take the time of the minimum temporal derivative of the signal[17]. This method can produce multiple peaks, making identifying an activation time unclear, and therefore subject to arbitrary criteria[18], [19]. The Laplacian reduces far field effects, emphasizing local activity. This technique has been particularly useful when attempting to identify activations of the conduction system in VF when the relative timing of the conduction system and the underlying VM activation are unknown. During sinus rhythm, activation propagates through the conduction system to the working myocardium, therefore, the conduction system activation temporally precedes the activation of the local VM. The zero crossing of the Laplacian occurs when the local tissue is transitioning from a current source to a current sink and therefore is considered the activation. The slope of the Laplacian is also at a maximum as the activation passes immediately beneath the central electrode of the Laplacian. By calculating the temporal derivative of the Laplacian, clear peaks can be distinguished which allows for a thresholding method to calculate an activation rate. The derivative of the Laplacian simplifies the detection of local activation time and provides some immunity to noise inherent in these signals.

During perfused VF the activation rate of the VM is significantly higher than the His bundle. This is consistent with the traditional theory that VF is maintained through rapid reentrant wavefronts within the VM[20]–[22]. However after 1–2 minutes of VF a transition occurs in which the His bundle activates faster than the VM. The VM activation rate decreases quickly during VF while the His bundle activation rate does not significantly decrease. It is possible that the activity in the conduction system may be overdrive suppressed by the rapid activation rate in the VM, and that the conduction system does not drive VF activation until the VM rate has slowed below the inherent activation rate of the conduction system. This finding suggest that the proximal conduction system may be an important driver of VF>2 minutes and is consistent with a more recent theory that the ventricular conduction system may have a critical role in VF maintenance, particularly after the first couple of minutes[10], [18], [23]–[26]. Three-dimensional simulations of the ventricles that attempt to include the conduction system suggest that the ventricular conduction system may be an important source for focal activity and may have a critical role in the development and sustaining of reentry during VF[27], [28]. A recent study demonstrated that ablation of the purkinje fibers slowed the activation rate during prolonged VF, and VF spontaneously terminated earlier than in control hearts[11]. This observation supports the simulation data that the ventricular conduction system may be required to maintain long duration VF.

Time may have a significant effect on the VM but not on the His bundle due to several mechanisms. First the His bundle has less metabolic load than the VM and therefore may take longer to deplete its reserved resources[29]. Second, much of His-Purkinje system is located directly on the endocardial surface and it may receive substantial nutrients from the fluid in the LV cavity. Our preliminary results suggested that if the electrode plaque was left on the His bundle for extended periods (>1 min), the His bundle developed slowed conduction or even blocked (unpublished data). Finally differences in ion channel kinetics and restitution properties of the ventricular conduction system and VM[26], may be responsible for the observed differences in the His and VM activation rate as a function of time.

The His bundle may have a slower activation rate than the VM during early VF but more rapid activation rate during prolonged VF due to several mechanisms. During sinus rhythm the VM has a shorter refractory period than the conduction system therefore electrical wavefronts on the myocardium may not be able to activate the conduction system at such a rapid rate [29]. As VF progresses and ischemic sets in, several electrophysiological changes occur in both the ventricular conduction system and the VM. One change is that the VM develops a longer refractory period than the conduction system [29] and the VM cannot maintain as high of activation rate. Another change during ischemic conditions is that distal purkinje fibers are thought to develop abnormal automaticity and/or triggered activity during ischemic conditions [6], [12], [26]. Triggered or abnormal automaticity in the distal purkinje fibers may conduct to the His bundle but block at the VM, due to several reasons. The first reason is due to the longer refractory period of the VM as compared to the conduction system. Second, there is a source-sink mismatch between the distal purkinje fibers and the VM[30]–[32]. This mismatch results in conduction occurring more readily from VM to distal purkinje fibers than from distal purkinje fibers to the VM[31]. The combination of longer refractory period of the VM as compared to the ventricular conduction system and the presence of a source sink mismatch between the distal purkinje fibers and the VM could result in a higher activation rate in the His bundle than the VM. Reentry involving both the VM and the conduction system [27] is another mechanism that could cause a more rapid activation rate in the ventricular conduction system. A single ventricular wavefront may conduct into the ventricular conduction system to the His bundle. Due to the limited access sites into the conduction system and the fast conduction velocity, a single VM wavefront, either propagating on the VM or which propagated through the conduction system, may activate the conduction system multiple times.

The His bundle conduction velocity reported for sinus rhythm in this study of 0.46±0.08 m/s (n = 10) was similar to previous reported ventricular conduction system velocities of 0.60±0.09 m/s (n = 3)[33]. The direction of conduction in the His bundle was nearly entirely retrograde during VF, consistent with activation propagating from the distal purkinje fibers through the bundle branches back to the His bundle. Antegrade conduction in the His bundle may only be observed if activation emerged from the AV node or through automaticity or triggered activity in the His bundle itself. However novel to this study was recording changes in His conduction velocity during the time-course of VF. Conduction in VF was slower for perfused VF with a normalized conduction velocity of 94% of sinus rhythm, which gradually decreased to 67% of sinus rhythm conduction at 8 minutes of VF. The initial 6% decrease in conduction velocity from sinus to perfused VF may due to the slower retrograde than antegrade conduction in the ventricular conduction system[34] or slowing associated with the higher activation rate in VF[35] due to conduction velocity restitution[34]. The decrease in conduction velocity during the time course of VF is most likely due to a combination of ischemia and a rise in resting potential and inactivation of Na+ channels during prolonged VF[35].

Recently published studies support the hypothesis that the distal conduction system is responsible for driving the rapid activation rate during long duration VF[10], [18], [23]–[26]. Studies of long duration VF have shown that activation patterns of the Purkinje system and working myocardium often propagate in patterns similar to the spread of activation through the conduction system during sinus rhythm[10], [36]. This work is the first to show direct evidence that the proximal ventricular conduction system activates more rapidly than the underlying working myocardium during long duration VF. The ventricular conduction system has a much faster conduction velocity than the VM, and only interacts with the VM at limited sites known as PMJs which are located only in the distal regions of the ventricular conduction system[13]. Therefore it is likely that once a distal Purkinje fiber is activated, it will quickly activate a large region of the conduction system, and as this study suggests, even conduct to the His bundle. The limited number of PMJs located only in the distal regions of the ventricular conduction system, in combination with the fast conduction velocity of the conduction system suggest that it is unlikely that activations through the ventricular conduction system will collide and block with other activations within the conduction system before activating large portions of the conduction system. Therefore it is likely that triggered activity in Purkinje fibers can conduct through the ventricular conduction system and drive rapid activation at distant sites, even opening the possibility of biventricular communication through the bundle braches.

### Limitations

A rabbit VF model was used for our study of properties of the His bundle during VF. VF is more difficult to initiate and maintain in rabbits than larger hearts such as humans. While it was difficult to initiate VF in rabbits, once it initiated, it was sustained for extended periods of time (with two exceptions). Another limitation is the use of an isolated heart preparation. In this preparation, the autonomic nervous system is severed which may influence VF. Due to these limitations the results of these findings may not directly translate to humans. In spite of these limitations, the isolated rabbit heart model is widely used and well accepted for studying VF[37]–[39]. A final limitation is that we mapped only a small region of the VM near the His bundle and atrial tissue therefore there may be regions of faster VM activation that were not a measured.

## Conclusion

In early VF (perfused VF), the VM has a faster activation rate than the His bundle. We concluded that the VM is likely the major driver of early VF, with the conduction system being driven by the activation of the VM. After 3 minutes of VF, the activation rate of the His bundle is faster than that of the VM. This observation suggests that activations in the distal ventricular conduction system propagate through the conduction system to activate the His bundle and may drive activation at distant sites. This finding supports the conclusion that the conduction system plays a critical role in the mechanisms of prolonged VF maintenance and may be a target for interventional therapy.
